# The Specific Carbohydrate Diet and Diet Modification as Induction Therapy for Pediatric Crohn’s Disease: A Randomized Diet Controlled Trial

**DOI:** 10.3390/nu12123749

**Published:** 2020-12-06

**Authors:** David L. Suskind, Dale Lee, Young-Mo Kim, Ghassan Wahbeh, Namita Singh, Kimberly Braly, Mason Nuding, Carrie D. Nicora, Samuel O. Purvine, Mary S. Lipton, Janet K. Jansson, William C. Nelson

**Affiliations:** 1 Department of Pediatrics, Division of Gastroenterology, Seattle Children’s Hospital and University of Washington, Seattle, WA 98105, USA; dale.lee@seattlechildrens.org (D.L.); ghassan.wahbeh@seattlechildrens.org (G.W.); namita.singh@seattlechildrens.org (N.S.); Kimberly.braly@seattlechildrens.org (K.B.); mason.nuding@seattlechildrens.org (M.N.); 2 Biological Sciences Division, Pacific Northwest National Laboratory, Richland, WA 99354, USA; young-mo.kim@pnnl.gov (Y.-M.K.); carrie.nicora@pnnl.gov (C.D.N.); janet.jansson@pnnl.gov (J.K.J.); william.nelson@pnnl.gov (W.C.N.); 3 Environmental Molecular Sciences Laboratory, Pacific Northwest National Laboratory, Richland, WA 99354, USA; samuel.purvine@pnnl.gov (S.O.P.); mary.lipton@pnnl.gov (M.S.L.)

**Keywords:** inflammatory bowel disease, Crohn’s disease, specific carbohydrate diet, nutrition, microbiome, multi-omics application

## Abstract

Background: Crohn’s disease (CD) is a chronic inflammatory intestinal disorder associated with intestinal dysbiosis. Diet modulates the intestinal microbiome and therefore has a therapeutic potential. The aim of this study is to determine the potential efficacy of three versions of the specific carbohydrate diet (SCD) in active Crohn’s Disease. Methods: 18 patients with mild/moderate CD (PCDAI 15–45) aged 7 to 18 years were enrolled. Patients were randomized to either SCD, modified SCD(MSCD) or whole foods (WF) diet. Patients were evaluated at baseline, 2, 4, 8 and 12 weeks. PCDAI, inflammatory labs and multi-omics evaluations were assessed. Results: Mean age was 14.3 ± 2.9 years. At week 12, all participants (n = 10) who completed the study achieved clinical remission. The C-reactive protein decreased from 1.3 ± 0.7 at enrollment to 0.9 ± 0.5 at 12 weeks in the SCD group. In the MSCD group, the CRP decreased from 1.6 ± 1.1 at enrollment to 0.7 ± 0.1 at 12 weeks. In the WF group, the CRP decreased from 3.9 ± 4.3 at enrollment to 1.6 ± 1.3 at 12 weeks. In addition, the microbiome composition shifted in all patients across the study period. While the nature of the changes was largely patient specific, the predicted metabolic mode of the organisms increasing and decreasing in activity was consistent across patients. Conclusions: This study emphasizes the impact of diet in CD. Each diet had a positive effect on symptoms and inflammatory burden; the more exclusionary diets were associated with a better resolution of inflammation.

## 1. Introduction

Crohn’s disease (CD), a subtype of inflammatory bowel disease (IBD), is an immune mediated inflammatory disorder of the gastrointestinal tract [1]. While the etiology has not been fully elucidated, CD is considered to be an immune dysregulation with an immune system activation and upregulation triggered by environmental insults and changes to the intestinal microbiome. As of 2015, about three million adults in the United States carry the diagnosis of IBD [2]. This rapid increase from approximately two million adults in 1999 emphasizes the likely role of environmental influences on the incidence of this disease [2]. Despite the role of environment, current IBD therapeutics concentrate on the paradigm of altering immunological functioning, with far less emphasis on the intestinal microbiome and mucosal barrier function [3]. 

The primary focus of therapy for inflammatory bowel disease is to directly suppress the immune system’s ability to activate and sustain an inflammatory response. While this approach can be effective, it does not reliably induce and maintain remission for every patient and is associated with significant side effects and costs. Given the incomplete efficacy of medication therapy, as well as the continuing rise of healthcare cost, interest in the role of environmental impacts on disease has emerged with a focus on diet. Dietary manipulation affects IBD via a mechanism of action that is uniquely different from current medication therapies. The primary impact of dietary therapy is on the intestinal microbiome and mucosal integrity with subsequent anti-inflammatory effects [4].

Diet shapes the composition of the intestinal microbiome [5]. In a murine model of mice deficient of genes relevant to host-microbial interactions (MyD88 -/-, NOD2 -/-, ob/ob and Rag1 -/-) and >200 outbred mice, diet reproducibly altered the gut microbiota despite differences in host genotype [6]. In humans, environmental influences, including diet, have been shown to be the major determinants of the intestinal microbiota and dominate over genetics in shaping human intestinal microbiota [7]. Diet also affects mucosal integrity [6,8]. A Western diet rich in fats and simple sugars in CEABAC10 mice disrupts host barrier functions, leading to thinning of the mucus layer and increase mucosal permeability [9]. Common dietary additives, such as emulsifiers, carboxymethylcellulose (CMC), maltodextrin and polysorbate-80 (P80), also increase intestinal permeability, erode the mucous layer, and promote a robust colitis in genetically predisposed mice [10,11,12]. 

The impact of diet in clinical medicine has best been shown with exclusive enteral nutrition (EEN) where it is utilized as first line therapy in pediatric CD [13,14,15,16,17]. Many whole food diets have been reported to be efficacious as well, but with less rigorous study [18]. These diets are an area of intense research given the potential to improve patient outcomes and decrease the overall cost of healthcare. Multiple dietary interventions have been noted to have a benefit in IBD, including the Crohn’s disease exclusion diet (CDED) and the Specific Carbohydrate Diet (SCD) [19,20]. The SCD was created by Sydney Haas MD, an academic pediatrician in 1924 to treat celiac disease [21]. It was then later used for the treatment of IBD [22]. The SCD eliminates all grains, sugars (except for honey), all milk products (except for hard cheeses and yogurt fermented 24 h) and most processed foods [23]. The presumed mechanism(s) of action of the SCD in IBD is through shifting of the intestinal microbiome as well as through improvement in mucosal integrity. 

Preliminary studies of the SCD have shown promising results [20,24]. Both clinical remission and normalization of inflammatory markers have been observed without the addition of medication therapy in active CD and ulcerative colitis (UC) [20,24]. To evaluate the potential mechanism of action of diet as well as the clinical impact of the SCD and two modified versions of this exclusionary diet, we initiated a double-blind diet-controlled study in active Crohn’s disease with a focus on clinical and laboratory parameters as well as the evaluation of the gut microbiome. We used a multi-omics approach to analyze stool samples collected during the trial, including microbial community profiling (16S ribosomal RNA amplicon sequencing), community DNA sequencing (metagenomics), expressed proteins as a measure of function (metaproteomics), and metabolomics to define the metabolic signatures of the dietary response.

## 2. Materials and Methods

### 2.1. Study Setting and Participants

This single center, double-blind study to determine tolerability, and efficacy of dietary therapy in pediatric patients with CD was conducted in the outpatient pediatric gastroenterology center. Patients aged 7 to 18 years with mild to moderate CD, as defined by a Pediatric Crohn’s Disease Activity Index (PCDAI) score of 15–45, were eligible to enroll. Diagnosis of Crohn’s disease was made by a primary gastroenterologist based on history, physical exam, laboratory/radiological studies, endoscopic evaluation and gastrointestinal histology. Patients must not have had medication changes for his/her inflammatory bowel disease medications for at least two months prior to enrollment. Exclusion criteria included active or a history of intraabdominal abscess, perianal abscess, perianal fistula, intraabdominal fistula, stricturing Crohn’s disease or other serious medical conditions such as neurological, liver, kidney, autoimmune or systemic disease. 

The protocol was approved by the Institutional Review Board at Seattle Children’s Hospital (IRB#15606). The study was registered with *ClinicalTrials.gov *(NCT02610101). All patients/participants provided written informed consent or assent.

### 2.2. Randomization and Blinding

The data was recorded and stored in Redcap. Patients were recruited and followed from November 2015 to December 2018. Block randomization of 1:1:1 was performed in blocks of three. Randomization was generated by REDCap, initiated after patient enrollment. The study coordinator shared the randomization information with the study chef to facilitate the correct meal plan. The principle investigator, study physicians/dieticians, study participants, and participants parents/guardian were blinded from the results of the diet randomization.

#### Study Intervention

**Figure 1:**
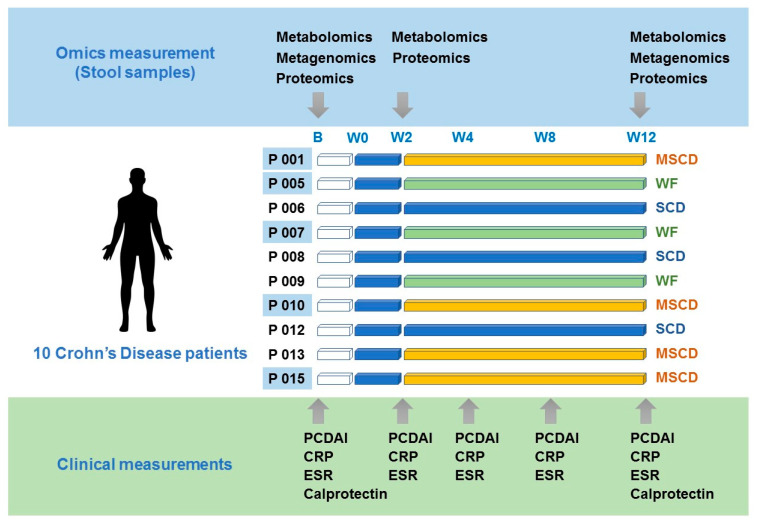
Experimental Design of Blinded Diet Controlled Study in Active Crohn’s Disease. Patients followed from Baseline visit (B) to Week 12 (W12) at specified time points with clinical assessment (PCDAI; Pediatric Crohn’s Disease Activity Index), laboratory assessment (C-reactive protein, sedimentation rate, complete blood count, and calprotectin) as well as metagenomics, metabolomics, and proteomic assessment (in highlighted patients: P001, P005, P007, P010, and P015). Specified diets are denoted as SCD (Specific Carbohydrate diet; blue), MSCD (Modified Specific Carbohydrate diet; yellow) and WF (Whole foods; green).

Patients were randomized via Redcap to one of three diets for 12 weeks: the SCD, SCD with oats and rice (modified SCD, MSCD), or a whole food diet (WF) eliminating wheat, corn, sugar, milk and food additives (Figure 1). For the first two weeks, all patients went onto a strict SCD after and were placed onto their randomized diet plan. Patients received one-on-one education and counseling by a dietitian trained in the SCD. Prior to each visit, patients completed a three day food intake record to help assure compliance with the diet. Patient meals were provided and prepared by a study chef. In addition, patients received a list of SCD approved foods allowed during the study. Patients followed-up and were in contact with the dietitian, research assistant and primary gastroenterologist for questions and the problem intervention with the diet over the 12-week study. 

### 2.3. Assessment of Participants

Study subject initial evaluation included history, physical exam, and lab tests, including complete blood count (CBC) with differential, C-reactive protein (CRP), erythrocyte sedimentation rate (ESR), albumin, stool for infectious work-up (*C. difficile*, bacterial pathogen culture and ova and parasites) was performed, as well as stool calprotectin and multi-omics analysis.

Patients had clinical follow-up at 2, 4, 8, and 12 weeks (Figure 1). Standardized questionnaires, including the PCDAI, were completed during each study visit [25]. In addition, patients had a physical exam and standard blood work including CBC, sedimentation rate, C-reactive protein, albumin and stool for multi-omics analysis at each follow up visit. Measurement of stool calprotectin was repeated at week 12. Descriptive statistics were generated, including mean, standard deviation, median, and range for continuous variables and frequency for categorical variables.

### 2.4. Metagenomics

‘Omics data was collected for five patients (P001, P005, P007, P010, and P015). Stool samples taken at enrollment (baseline) and at the end of the treatment course (week 12) underwent shotgun metagenomic sequencing. Sequencing was performed by GeneWiz, Inc. (South Plainfield, NJ, USA). Libraries were prepared using AllPrep PowerViral DNA/RNA Kit (Qiagen), and samples were multiplexed into two lanes of Illumina HiSeq 2 × 150 bp sequencing, yielding 130–170 million reads per sample. Raw reads were trimmed and screened for the adapter sequence using bbduk [26]. Kraken2 and the Kraken2_std reference database [27] were used to assign taxonomy to unassembled reads. For each patient, baseline and week 12 datasets were co-assembled using megahit v1.2.5 [28]. Contigs longer than 2500 bp were used to develop reference protein datasets for proteomics analysis. Protein-coding genes were predicted using prodigal [29] and functional annotation was assigned to the predicted proteins using Prokka [30]. Read coverage of scaffolds was calculated by searching the quality-trimmed read sets against the scaffold sets using bwa-mem2, samtools, and mosdepth [31,32,33].

### 2.5. Protein/Metabolite Extraction

Fecal samples from five patients (three whole food diets and two modified SCD) were obtained prior to adopting the diet (baseline) and at 2 and 12 weeks afterwards (Figure 1). Samples were lyophilized and physically ground. Equal dry weight amounts were then transferred to a chloroform compatible microcentrifuge tubes for metabolite and protein extraction. The average weight was 20.08 ± 1.33 mg. A solvent mixture of chloroform/methanol/water was used to extract metabolites, as reported previously [34]. This results in two solvent layers, including an upper aqueous phase (containing hydrophilic metabolites) and a lower organic phase (containing lipids and other hydrophobic metabolites), while proteins precipitate in the interphase. 

### 2.6. Metaproteomics

***Tryptic Digestion***. The protein interlayer underwent tryptic digestion as previously described [35]. Briefly, the fraction was washed with methanol, pelleted and dried. Pellets were resuspended in 8M urea. A reducing agent, dithiothreitol, was added to 10 nM and samples were incubated at 60 °C for 30 min. Samples were diluted 8-fold with 100 mM NH_4_HCO_3_, 1 mM CaCl_2_, and sequencing-grade modified porcine trypsin (USB, Santa Clara, CA, USA) was added to a 1:50 (*w*/*w*) trypsin-to-protein ratio and incubated for 3 h at 37 °C. Digested samples were desalted using a 4-probe positive pressure Gilson GX-274 ASPEC™ system (Gilson Inc., Middleton, WI, USA). Samples were eluted with 1 mL 80:20 ACN:H2O, 0.1% TFA. The samples were concentrated and 250 µg of peptide from each sample was collected for high performance liquid chromatography (HPLC fractionation). 

***HPLC***. High performance liquid chromatography was performed as previously described [ibid]. Briefly, samples were resolved on a XBridge C18, 250 × 4.6 mm, 5 μM with 4.6 × 20 mm guard column (Waters, Milford, MA, USA) using an Agilent 1100 series HPLC system (Agilent Technologies, Santa Clara, CA, USA) with mobile phases (A) 10 mM ammonium formate, pH 10.0 and (B) 10 mM ammonium formate, pH 10.0/acetonitrile (10:90). A total of 96 fractions were collected. Every 8th fraction was combined for a total of 12 samples (each with n = 8 fractions pooled). Fractions were dried down, resuspended to 0.1 µg/µL and stored at −20 °C until LC-MS/MS analysis.

***Mass Spectrometry***. All data were collected on a Q Exactive mass spectrometer (Thermo Electron, Waltham, MA) coupled to a Thermo/Dionex Ultimate 3000 high performance liquid chromatography through 75 μm × 74.5 cm columns packed with Phenomenex Jupiter C-18 derivatized 3 um silica beads (Phenomenex, Torrance, CA, USA). Samples were loaded onto columns with 0.05% formic acid in water and eluted with 0.05% formic acid in Acetonitrile over 120 min. Twelve centroided data-dependent HCD MS/MS scans were recorded for each survey MS scan (35 K nominal resolution) using normalized collision energy of 30, isolation width of 2.0 m/z, and rolling exclusion window lasting 30 s before previously fragmented signals are eligible for re-analysis. 

***MS/MS data search***. The MS/MS spectra were interpreted for charge and parent mass values using MSConvert [36]. MSGFPlus [37] was used to assign spectra to peptides (+/− 20 ppm parent mass tolerance, partial tryptic enzyme settings, and a variable posttranslational modification of oxidized methionine). A target-decoy approach [38] was employed, first with each sample searched against its specific metagenome, then against the 2019_2 Uniprot SPROT release for *Homo sapiens*, both combined with typically observed contaminant proteins (keratins, trypsin, etc.). 

***Data analysis***. Results were filtered to below 1% FDR using an MSGF+ supplied Q-Value that assesses reversed sequence decoy identifications for a given MSGF score across each dataset. Filtered metagenome results were then combined with >1% filter passing results from the *H. sapiens* search at the MS2 spectrum level, using the MSGFScore value to determine which identification was to be used in the final results. Using the protein references as a grouping term, unique peptides belonging to each protein were counted, as were all PSMs belonging to all peptides for that protein (i.e., a protein level observation count value). All proteins belonging to the grouped peptides were reported along with their grouped descriptions. Protein level PSM observations were enumerated for each sample, allowing low-precision quantitative comparisons to be made. Only proteins supported by at least two peptides were considered in this analysis.

### 2.7. Metabolomics

To examine volatile metabolites, 50 µL of the aqueous portion was directly transferred to new vial with the same volume of methanol and analyzed by gas chromatography–mass spectrometry (GC-MS) with a polar column (FFAP-Free fatty acid phase separation). The samples for the volatile metabolites were strictly temperature controlled at −20 °C until the analysis to minimize the environmental effect. The remainder of the aqueous polar layer was combined with the organic layer and dried using a speed-vacuum concentrator. The samples were subsequently subjected to a two-step chemical derivatization process and analyzed by GC-MS analysis with a non-polar separation column, HP-5MS. The collected raw GC-MS data were processed by Agilent Chemstation and Metabolite Detector programs to identify metabolites by searching against metabolomics databases (PNNL in-house developed one, and NIST17/Wiley 11) [39].

### 2.8. Data Analysis 

Richness calculations, inverse Simpson diversity analysis and NMDS analysis were performed using the vegan package [40] in R (https://www.r-project.org/). The small sample sizes for the ‘omics datasets prohibited robust determinations of statistical significance, so a heuristic was used to screen for consistent changes in protein expression abundance. The identified proteins were grouped by assigned Enzyme Classification (EC) identifiers, and relative abundances were calculated using the summation of peptide counts of group members as an estimation of abundance. The dataset was screened for enzyme classes that (1) either increased or decreased at least 2-fold between two timepoints being compared in at least one of the patients in the treatment group, (2) did not show an opposite 2-fold change in any other patient in the group, and (3) had a mean fold-change across all patients in the group with a magnitude larger than the standard deviation. It is important to note that non-enzyme proteins and enzymes that were not assigned an EC number by the underlying annotation protocols were not examined.

## 3. Results

### 3.1. Participant Characteristics 

**Figure 2:**
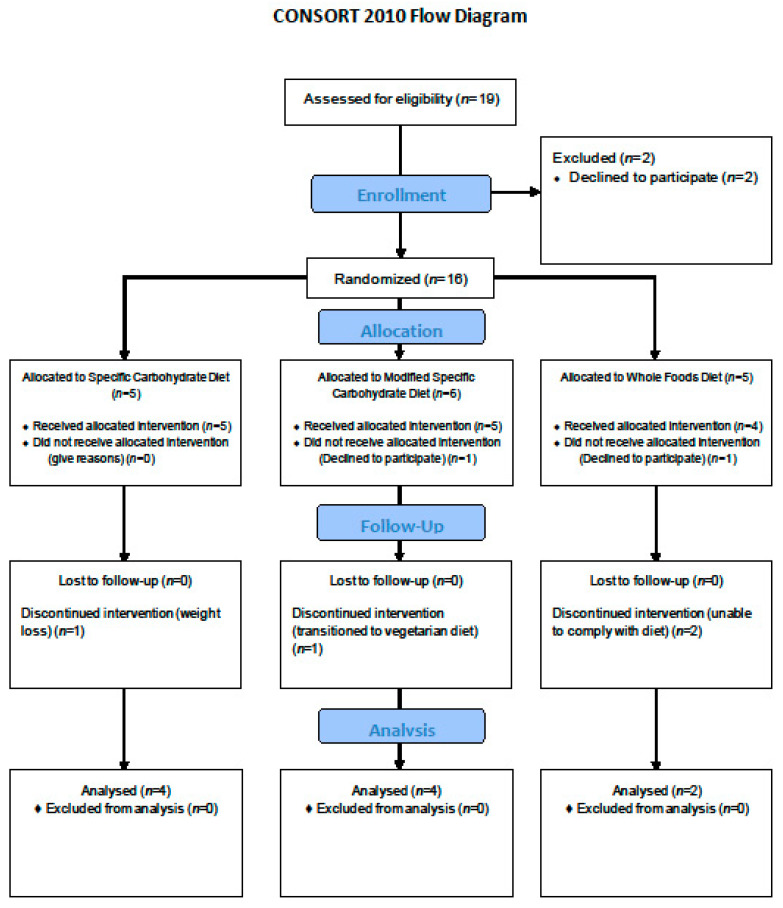
Consort Flow Diagram.

Eighteen individuals were enrolled. Ten participants completed the study (Figure 2). Four patients stopped the study prior to completion (two participants completed up to W4. Two participants completed up to W8). Two patients were screen failures and were not randomized. Two completed only the screening visit and decided not to participate due to the complexities of the diet. 

Mean age was 14.3 ± 2.9 years (range 7–18 years). 10 Males (55%) were enrolled. Macroscopic disease at time of diagnosis per Paris classification was as follows: ileal (L1) in one patient, colonic (L2) in three patients, and ileocolonic (L3) in 14 patients.

At the time of entrance into the study, patients indicated taking aminosalicylates (n = 4), immunomodulators (n = 3; azathioprine (1), 6-mercaptopurine (1), methotrexate (1)), biologics (n = 4; infliximab (4)), and no medication (n = 8) (Table 1).

### 3.2. Clinical Outcomes 

Two weeks after initiation of the dietary therapy, nine of the fourteen patients were in clinical remission based on PCDAI scoring, defined for PCDAI as less than ten. At week 4, nine of the remaining 13 participants achieved clinical remission. At week 12, all participants (n = 10) who completed the study achieved and maintained clinical remission. In subgroup analysis, the PCDAI improved with in each group. 

**Figure 3:**
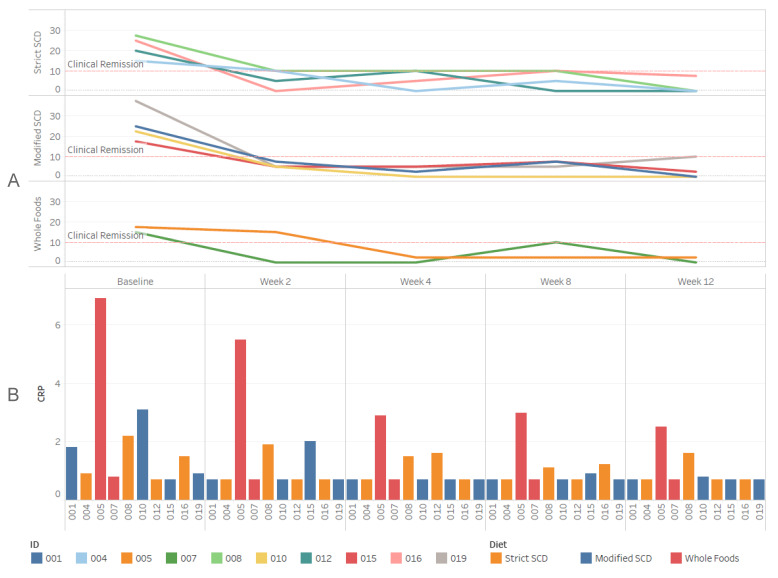
Clinical and laboratory outcomes during dietary therapy. (**A**) Pediatric Crohn’s disease Activity index per dietary therapy (SCD, MSCD, and WF) over 12 week study period (**B**) C-reactive protein per patient per dietary therapy (orange *=* SCD; blue *=* MSCD; red *=* WF) over 12 week study period.

Two weeks after initiation of the dietary therapy, nine of the thirteen patients with elevated CRP had improved their CRP. By week 12, all eight participants who had elevated CRP at enrollment and completed the study had normal CRP (Figure 3). In subgroup analysis, the CRP improved within each group with normalization of CRP in the SCD and MSCD groups. Two weeks after initiation of the dietary therapy, 10 of the fourteen patients showed a decrease in their ESR. By week 12, eight out of ten participants who completed the study had a decrease in ESR. In subgroup analysis, the ESR improved within each group with normalization of ESR in the SCD and MSCD groups.

### 3.3. Specific Carbohydrate Diet

By Intention to Treat (ITT) analysis, the PCDAI decreased from 23.5 ± 6.0 at enrollment to 14 ± 17.8 at 2 weeks and 1.9 ± 3.8 at 12 weeks in the SCD group (Figure 3). By the Per Protocol (PP) analysis, the PCDAI decreased from 21.9 ± 5.5 at enrollment to 6.25 ± 4.8 at 2 weeks and 1.9 ± 3.8 at 12 weeks. The CRP by ITT analysis decreased from 1.3 ± 0.6 mg/dL at enrollment to 1.1 ± 0.6 mg/dL at 2 weeks and 0.9 ± 0.5 mg/dL at 12 weeks. The CRP by PP analysis decreased from 1.3 ± 0.7 mg/dL at enrollment to 1.0 ± 0.6 mg/dL at 2 weeks and 0.9 ± 0.5 mg/dL at 12 weeks. The ESR by ITT analysis decreased from 28.0 ± 30.9 mm/h at enrollment to 22.8 ± 33.9 mm/h at 2 weeks and 13.0 ± 14.2 mm/h at 12 weeks. The ESR by PP analysis decreased from 15.6 ± 13.7 mm/h at enrollment to 7.8 ± 4.4 mm/h at 2 weeks and 13.0 ± 14.2 mm/h at 12 weeks. The calprotectin did not change statistically in the SCD group from 350 ± 271 mg/kg at enrollment to 572 ± 605 mg/kg at 12 weeks.

### 3.4. Modified SCD

ITT analysis for the MSCD group showed a decreased PCDAI from 29.6 ± 9.5 at enrollment to 8.0 ± 5.4 at 2 weeks and 3.1 ± 4.7 at 12 weeks. PP analysis for the MSCD group showed a decreased PCDAI from 25.6 ± 8.5 at enrollment to 5.6 ± 1.3 at 2 weeks and 3.1 ± 4.7 at 12 weeks. The CRP by ITT analysis decreased from 3.5 ± 5.2 mg/dL at enrollment to 1.0 ± 0.6 mg/dL at 2 weeks and 0.7 ± 0.1 mg/dL at 12 weeks. The CRP by PP analysis decreased from 1.6 ± 1.1 mg/dL at enrollment to 1.0 ± 0.7 mg/dL at 2 weeks and 0.7 ± 0.1 mg/dL at 12 weeks. The ESR by ITT analysis decreased from 42.0 ± 26.8 mm/h at enrollment to 20.2 ± 14.9 mm/h at 2 weeks and 14.2 ± 10.2 mm/h at 12 weeks. The ESR by PP analysis decreased from 32.3 ± 10.2 mm/h at enrollment to 19.0 ± 17.0 mm/h at 2 weeks and 14.3 ± 10.2 mm/h at 12 weeks. The calprotectin decreased from 697 ± 520 mg/kg at baseline to 157 ± 156 mg/kg at 12 weeks in the MSCD group.

### 3.5. Whole Food

ITT analysis for the WF group showed a decreased PCDAI from 21.6 ± 8.4 at enrollment to 15.0 ± 10.8 at 2 weeks and 1.3 ± 1.8 at 12 weeks. PP analysis for the MSCD group showed a decreased PCDAI from 16.25 ± 1.8 at enrollment to 7.5 ± 10.6 at 2 weeks and 1.3 ± 1.8 at 12 weeks. The CRP by ITT analysis decreased from 3.2 ± 2.6 mg/dL at enrollment to 2.5 ± 2.2 mg/dL at 2 weeks and 1.6 ± 1.3 mg/dL at 12 weeks. The CRP by PP analysis decreased from 3.9 ± 4.3 mg/dL at enrollment to 3.1 ± 3.4 mg/dL at 2 weeks and 1.6 ± 1.3 mg/dL at 12 weeks. The ESR ITT analysis decreased from 21.2 ± 16.0 mm/h at enrollment to 21.0 ± 21.3 mm/h at 2 weeks and 15.5 ± 14.9 mm/h at 12 weeks. The ESR by PP analysis decreased from 27.0 ± 29.7 mm/h at enrollment to 28.0 ± 33.9 mm/h at 2 weeks and 15.5 ± 14.9 mm/h at 12 weeks. The calprotectin decreased from 740 ± 696 mg/kg at enrollment to 420 ± 166 mg/kg at 12 weeks whole foods group.

Adverse events likely related to diet in the study included weight loss (n = 1), lethargy (n = 2), hunger (n = 1) and allergic reaction (n = 1, split pea flour). Adverse reactions possibly related to diet include abdominal pain (n = 4). Adverse events unlikely related to dietary intervention include upper respiratory illness (n = 3) and acne (n = 1).

### 3.6. Microbiome

#### 3.6.1. Metagenomics

**Figure 4:**
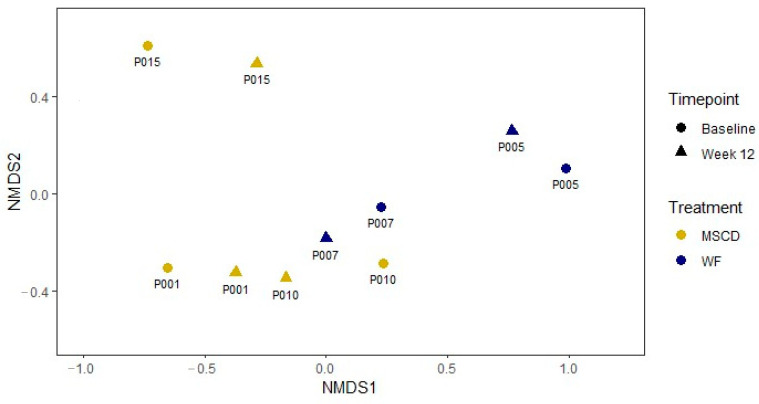
Microbiome community structure NMDS. Taxonomy of individual reads, as assigned by Kraken2, was compared between timepoints (baseline and week 12 only). Non-metric multi-dimensional scaling was used to plot the data. The individuality of each patient’s microbiome is evident from patient-specific distances being closer than treatment-specific or timepoint-specific distances. The week 12 spread is less than the baseline spread, indicating more similar community composition following dietary treatment.

As expected, the microbiomes of the five patients were highly individual, however, each patient demonstrated a shift in community structure over the course of the treatment (Figure 4). Taxonomic analysis of the metagenomic reads revealed that the samples had a large percentage of unclassified reads (25%–50%), and while all samples yielded some human sequence, the MSCD005 enrollment sample contained almost 30% human sequence. For classified, non-human sequences, richness was maintained across the treatment period for all patients, and Inverse Simpson indexes showed an overall trend of increasing diversity with values ranging from 4.67 to 14.09 at enrollment and increasing to a range of 10.93 to 17.76 at the end of the study (Table 2). However, not all patients’ microbiomes increased in diversity. MSCD001 and MSCD010 both decreased modestly (13% and 19%, respectively), while the others increased. Patients MSCD0005 and MSCD015 had low diversity prior to adopting the dietary regime, MSCD005 being dominated by *Faecalibacterium prausnitzii* (~40% of classified bacterial reads) and MSCD015 being dominated by *Bacteroides ovatus* (~34%) and *Bacteroides xylanisolvens* (~13%) (Supplementary Materials Data 1). 

Over the course of the treatment, the abundance of some bacterial populations in each patient changed 10-fold or more. Organisms that increased in abundance in at least four of the five patients, including a *Blautia* species, a *Lachnospiraceae* species, *Faecalibacterium prausnitzii*, *Roseburia hominis*, *Roseburia intestinalis*, *Anaerobutyricum hallii*, and *Eubacterium eligens*. Organisms that decreased in more than three patients included *Escherichia coli* and a strain of *Faecalibacterium prausnitzii*.

#### 3.6.2. Metabolomics

*Volatile metabolite analysis*. We resolved 58 volatile metabolite peaks from the samples, of which 25 were able to be identified by comparison against reference databases. Principal component analysis (PCA) (Figure S1) showed that the overall profile of volatile metabolites changed over the treatment. The clustering of individual volatile metabolome profiles was centered after the 12th week of treatment, in comparison to the scattered profiles from the baseline samples. A significant decrease (n = 5, *p =* 0.00004) was observed for 1,2-proandiol from baseline to week 2, while glycerol increased (n = 5, *p =* 0.06257). The number of treatment-specific samples was not sufficient (MSCD n = 3, WF n = 2) for statistical analyses between samples from week 2 to week 12. However, the compounds 1,2-propanol, tetrahydro-2-pyranone, and 3-pyridol showed a trend of decrease over that time period, while acetate, pentanoate, and 2-phenylmalonic acid showed a trend of increase (Table 3). 

*Global metabolite analysis.* The global metabolite fractions presented very complicated metabolite profiles (~340 peaks resolved) from the stool samples; 169 peaks were identified through comparison against the reference database. PCA analysis showed a strong host-specificity and no apparent temporal effect. Comparison between the baseline and the week 2 samples, when all patients were following the SCD treatment, revealed 19 metabolites with significant changes in abundance (*p* ≤ 0.05) (Table 4). Sterol types of metabolites, such as campesterol and stimasterol, and maltose decreased; whereas some fatty acids, oleic acid and lignoceric acid, and several amino acid precursors, such as pimelic acid, increased. Abundance trends from weeks 2–12 also suggested that stigmasterol increased and pimelic acid decreased (although these trends were not significant), perhaps reflecting a normalization of microbiome activity following the disturbance caused by the dietary change.

#### 3.6.3. Metaproteomics Analysis

**Figure 5:**
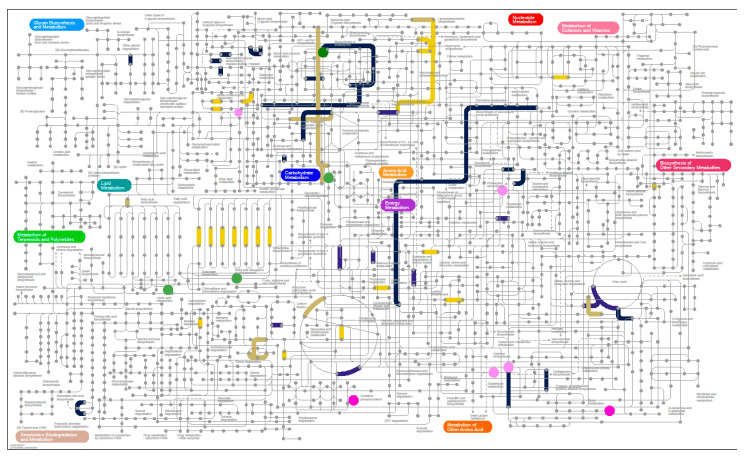
Functions and metabolites changing in abundance under the SCD diet. Proteomics results, grouped by EC classification, from week 2 samples were compared against baseline samples. Functions showing a more than 2-fold increase or decrease in at least one patient are highlighted. EMBL’s Interactive Pathways Explorer v3 (iPath3) was used for visualization (doi:10.1093/nar/gyk299). Metabolites that changed in abundance are also highlighted (circles). Navy blue, decreased abundance (*p* ≤ 0.05); tan, increased abundance (*p* ≤ 0.1); yellow, increased abundance (*p* ≤ 0.05). Interactive figures are available at https://pathways.embl.de/selection/wHoxRtfd7dXNlBRp2tz.

**Figure 6:**
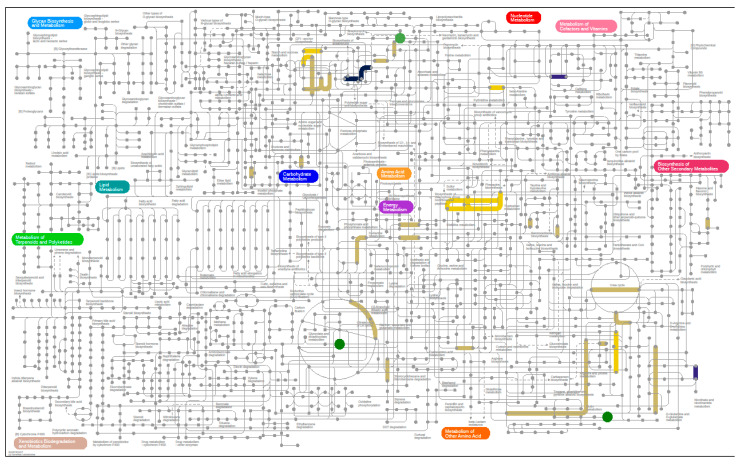
Functions and metabolites changing in abundance under the MSCD diet. For patients transitioned to the MSCD diet proteomics results, grouped by EC classification, from week 12 samples were compared against week 2 samples. Metabolites that increased over this time period are also highlighted (circles). Data is presented, as described in Figure 5. Interactive figures are available at https://pathways.embl.de/selection/9W7hzGUbabnlDYQQitH.

**Figure 7:**
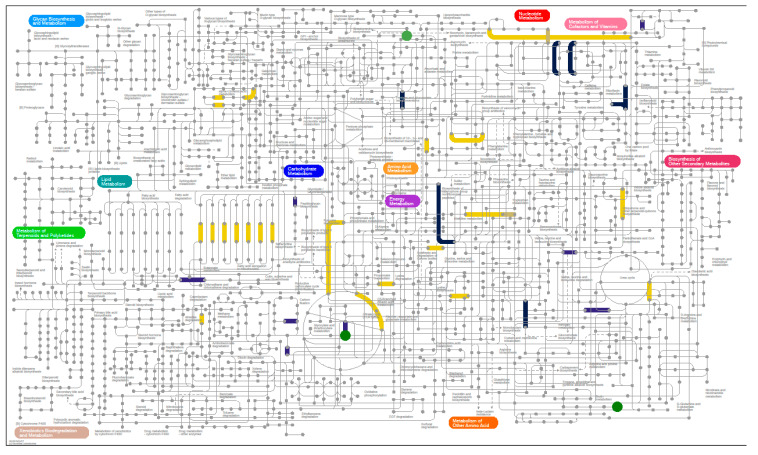
Functions and metabolites changing in abundance under the WF diet. For patients transitioned to the WF diet proteomics results, grouped by EC classification, from week 12 samples were compared against week 2 samples. Metabolites that increased over this time period are also highlighted (circles). Data is presented, as described in Figure 5. Interactive figures are available at https://pathways.embl.de/selection/lmukWBizoWD7FIQHot.

The community “metaproteome” protein fractions were highly complex, with between 14,643 to 27,954 protein identifications arising from the patients (Table 5). Due to the similarity of protein sequences among bacterial strains, it is common for peptides to map to multiple reference proteins. For this reason, nonunique peptides were mapped to a representative protein, and the identified proteins were further grouped by enzyme classification (EC) for comparison, thus mitigating the ambiguity of the peptide to protein matching. This approach revealed the dynamics of enzymatic functions rather than specific proteins. Changes in relative abundances of EC assignments between timepoints were assessed, and functions showing a greater than 2× difference in at least one patient, which were not contradicted in any other patient (i.e., showed a 2× difference in the opposite direction) were assessed for their metabolic role. Several general categories of metabolism appeared to be affected by the dietary intervention. These results demonstrate commonalities in the functional shift occurring in the luminal microbiome in response to the dietary change. Decreases in metabolic activities relating to starch breakdown and metabolism of simple sugars, in particular galactose, were observed (Figure 5). In addition, changes in the amino acid metabolism pathways suggested a shift from amino acid biosynthesis to catabolism. Increases in proteins involved in pentose and glucuronate interconversions (ECs 5.3.1.17, 5.3.1.14, 1.1.1.58, 4.2.1.7, and 4.1.2.19) could indicate increased metabolism of plant polysaccharides such as pectin [41]. The subsequent switch to either the MSCD or the WF diet resulted in fewer metabolic shifts, as might be expected since these dietary changes were less pronounced than the initial shift to the SCD (Figure 6 and Figure 7). Patients on the MSCD showed additional increases in amino acid metabolism. Increases in some functions involved in starch and sucrose metabolism could reflect the limited addition of carbohydrates to the diet. Patients on the WF diet also showed additional increases in amino acid metabolism and decreases in glutamate synthase (EC 1.4.7.1), methionine synthase (EC 2.1.1.13) and 2-dehydro-3-deoxy-D-pentonate aldolase (EC 4.1.2.28), which is involved in converting xylose to pyruvate. Other functions changed over time, but the change was inconsistent across patients. For example, proteins involved in histidine biosynthesis (ECs 2.4.2.17, 3.1.3.15, and 3.6.1.31) and the pentose phosphate pathway (ECs 1.1.1.44, 1.1.1.49, and 3.1.1.31) were observed to increase 2-fold in at least one patient and decrease in at least one other. 

## 4. Discussion

Crohn’s disease is the archetypal immune mediated disease connected to both the industrialization and westernization of the modern world. It is associated with changes in the intestinal microbiome and diet. We have shown that modification of the western diet is associated with clinical improvement as well as a decrease in inflammatory burden in CD. We present the first blind diet-controlled study of the specific carbohydrate diet compared to two liberalized versions, MSCD and WF. We show that all the diets were associated with high and comparable rates of clinical remission and all had improvement in inflammatory burden to differing degrees. While the MSCD group had normalization and the SCD group had near normalization of ESR and CRP, the whole foods group did not. The improvement in clinical and inflammatory burden may be associated with similarities in diet. A strict SCD removes all grains, milk products except for hard cheeses and a yogurt fermented for 24 h, sugars outside of honey and most processed foods. Similarly, the MSCD and WF diet removed simple sugars, lactose and highly processed foods. These excluded foods have been implicated in both the inflammation as well as intolerances. The difference seen in improvement in inflammatory markers based on CRP and ESR with the SCD and MSCD, as compared to the WF diet, may be related to degree of liberalization of diet itself.

While great advances have occurred in our understanding of disease pathogenesis, significant gaps still exist. The current understanding of the IBD paradigm focuses on the interaction between the immune system, intestinal barrier and the gut microbiome. In IBD, alteration or dysbiosis of the intestinal microbiome is thought to instigate and perpetuate a pro-inflammatory state that results in immune activation [3]. This divergence from a healthy individual’s intestinal microbiota is characterized by a decrease in commensal bacteria including members of Firmicutes and Bacteroides as well as a relative increase in pro inflammatory bacteria such as Enterobacteriaceae [42,43]. In addition, a decrease in butyrate producing bacteria, which are important in intestinal health, has been seen in patients with CD [44]. Despite the lack of clarity on the trigger of the dysbiosis, the mechanism by which dietary therapies are believed to have an effect is through manipulation of the intestinal microbiome with improvement in mucosal integrity [4]. In a previous prospective study of the SCD in pediatric IBD, Crohn’s patients with active disease had improved clinical disease activity indexes (PCDAI) with the majority of patients achieving clinical remission in conjunction with normalization/improvement in CRP and ESR over a 12-week period. Intestinal microbiome analysis of these patients showed a distinctive dysbiosis for each individual in most pre-diet microbiomes with significant changes in microbial composition after dietary change [20].

Building upon this prior study, here we confirmed clinical and biochemical improvement in patients on three distinct dietary therapies. Since all patients were initially treated with the SCD, we chose to perform the omics analysis on the patients which were switched to the MSCD and WF, so all dietary effects could be assessed. The observed improvement in clinical indices and biochemical markers was associated with changes in the intestinal microbiome, metabolome and proteome. Over the course of the dietary treatment, we observed a change in each patient’s microbiome composition. The changes were taxonomically inconsistent across patients, even when taking into account the different diets. As has been well established in other studies [45,46,47,48,49], microbiome composition was highly individualized, with all within-patient samples being more similar to each other than to other patients. Previous reports have described dysbiosis generally as including decreases in Bacteroidetes and Firmicutes [43,50,51,52]. Our patients, however, showed varying responses of these Phyla, with all the MSCD patients (P005, P007, P010) showing substantial increases in the relative abundance of Bacteroidetes largely at the expense of Firmicutes, one of the patients on the WF (P015) showing the opposite trend, and the other showing virtually no difference (P001). *Faecalibacterium prausnitzii*, a butyrate-producing bacterium which has specifically been called out in other IBD studies [53,54], varied between patients in both abundance and response to diet. However, it has also been previously reported that *F. prausnitzii* abundance differs between different IBD types; being particularly depleted in ICD and less so in CD or UC [44,49,54,55]. As we included patients that had inflammation at different intestinal regions, this could at least partly account for the different trends observed for *F. prausnitzii.* The consistent improvement of the health of the patients across all dietary treatments despite varying effects on microbiome community composition indicates that the microbiome metabolic activity is an important factor in disease progression.

Metaproteomic data revealed changes in carbohydrate metabolism and amino acid metabolism in response to the dietary treatments. These shifts in metabolic function are consistent with the reduction in simple and processed carbohydrates, causing the microbiome community to derive more of its energy from more complex nutrients. While there was evidence of an increase in utilization of complex carbohydrates such as pectin, decreases in the relative abundance of amino acid biosynthetic pathways could suggest either the increased salvage of amino acids for biomass production or the increased utilization of amino acids as an energy source. Approximately half of the metabolites we identified as changing significantly across the course of treatment were relevant to the same metabolic functions/reactions that were identified in our proteomics analysis. It is difficult to predict luminal metabolite levels from intestinal microbiome data since a metabolite could originate from the diet, host or microbiome activity, or a combination of all three, and the turnover rates of metabolites vary widely [56]. These data do demonstrate, however, that a multi-omics approach was capable of providing data that helped to elucidate the metabolic mechanism(s) by which nutritional therapy impacts the intestinal microbiome. Larger size of studies with appropriate controls should be used to further refine specific dietary effects on gut microbiome function, changes in the selective environment that lead to community structure shifts and resulting metabolite profiles that correlate with host health improvements.

In the evolving treatment paradigm of CD therapy, evidence of the impact of diet on inflammatory burden continues to grow. While the study of a whole foods diet in IBD is still in its scientific infancy, this study emphasizes the potential impact of diet in IBD and demonstrates the greater opportunity for physicians and researchers to further examine how to best address diet with IBD patients. While the results of this study suggest an overall benefit for patients on each of the three dietary therapy arms, there are significant limitations to this study. As an open-labeled study, recruited patients and parents may have had a strong personal belief that dietary change would improve symptoms. Thus, it cannot be excluded that participant bias could account for some of the effect seen in the PCDAI. Yet, objective markers of inflammation were found to decline with the interventions. While mucosal healing was not assessed directly with ileocolonoscopy and biopsy, fecal calprotectin is a validated surrogate marker for intestinal inflammation and was utilized for this study [57]. Another limitation to dietary therapy is the difficulty in ascertaining compliance with either the chef prepared foods or the diet as a whole. While we had studied participants’ meet with a dietitian at each visit to review the diet, there were no objective measures of dietary compliance. Finally, the small sample size for this pilot study limits the precision of estimated effects of the three dietary therapy arms for patients with IBD. The study itself also highlights some of the difficulty of research involving dietary therapy. Four individuals enrolled but withdrew from the study before making any dietary changes. Four individuals were unable to finish the study given difficulty maintaining the diet. This highlights the difficulty for patients to make significant, sustained dietary changes. Incentivizing patients by providing food may have resulted in the unintended consequence of selecting for some study participants not fully invested in making lifestyle changes, and thus not committed to strict compliance with a difficult study intervention. Despite these limitations, this study highlights the link between diet and IBD via the intestinal microbiota. Further studies of diet as a potential therapeutic option are required to better define mechanism(s) of action, maximize beneficial effects, and in the future tailor individualized therapy. As research in dietary intervention expands, it is imperative for researchers and clinicians to understand the complexity of dietary interventions and their associated lifestyle changes. By altering the intestinal microbiome, the purported trigger of the immune system, diet modification may have the ability to improve patient outcomes and the natural history of Crohn’s disease.

## Figures and Tables

**Table 1:** Reporting demographic variables of 10 patients in the study.

Participant Demographics		
**Anthropometrics**	Age (y)	14.4 ± 3.00
	Age at Diagnosis (y)	10.9 ± 4.83
	Height (cm)	158 ± 12.9
	Weight (kg)	52.2 ± 13.5
	BMI	20.5 ± 2.96
**Sex**	Male	8 (57.1%)
	Female	6 (42.9%)
**Disease Phenotype**	Inflammatory, non-penetrating, non-stricturing	14 (100.0%)
	Stricturing only	0 (0.0%)
	Penetrating only	0 (0.0%)
	Both stricturing and penetrating	0 (0.0%)
**Medications**	Aminosalicylates	4 (22.2%)
	Antibiotics for IBD	0 (0.0%)
	Biologics	4 (22.2%)
	Corticosteroids	0 (0.0%)
	Immunomodulators	3 (16.7%)
	Other immune suppresants	0 (0.0%)
	Rectal Therapy	0 (0.0%)
	None	8 (44.4%)
**PGA**	Normal	0 (0.0%)
	Mild	6 (42.9%)
	Moderate	8 (57.1%)
	Severe	0 (0.0%)

y: years.

**Table 2:** The richness and diversity of taxonomic assignments from the metagenomic analysis.

SaMPLE	Raw Reads	QC Reads	Unclassified ^a^	Human	Richness ^b^	Inv Simpson ^b^
**P001 B**	151,597,524	129,774,388	28.12%	1.75%	4218	14.05
**P001 12**	154,162,426	136,058,126	31.97%	0.30%	4218	12.14
**P005 B**	147,131,300	130,725,972	29.74%	32.42%	4216	4.64
**P005 12**	152,188,186	132,869,602	53.25%	1.28%	4216	10.86
**P007 B**	133,198,464	114,558,570	36.28%	0.26%	4215	13.57
**P007 12**	196,850516	172,507,988	34.46%	0.56%	4217	17.68
**P010 B**	148,457,710	123,323,294	30.41%	1.01%	4216	13.87
**P010 12**	160,592,310	150,922,746	25.34%	0.69%	4217	11.23
**P015 B**	150,180,158	110,457,378	32.76%	1.18%	4198	4.77
**P015 12**	165,946,068	130,556,042	47.70%	3.04%	4210	15.42

^a^. Taxonomy was assigned to individual reads using Kraken2 and the Kraken2_std reference database. ^b^. Only considering classifications to the species level and with an average abundance of ≥100 reads per sample and excluding human sequences.

**Table 3:** Volatile metabolites with large changes in abundance across treatment.

Metabolites	001		010		015		005		007	
	W02/S	W12/S	W02/S	W12/S	W02/S	W12/S	W02/S	W12/S	W02/S	W12/S
1,2-propanediol	11.5	6.7	15.4	8.6	10.5	6.2	39.7	26.7	21.0	12.8
Unknown 002	0.0	0.0	0.0	0.0	19.9	95.3	136.7	102.1	132.8	84.0
2-piperidinone	5.7	3.8	14.4	20.2	51.9	19.4	171.6	39.3	142.7	101.3
Unknown 018	66.4	3.8	6.0	19.8	108.7	84.2	244.0	195.1	97.8	89.6
benzenepropanoic acid	22.2	18.5	108.6	0.0	79.8	62.0	275.2	55.9	115.2	125.1
tetrahydro-2-pyranone	21.6	14.3	109.7	57.4	85.6	14.0	490.7	99.8	231.6	198.3
Unknown 010	68.8	48.6	81.8	65.1	44.2	23.2	160.7	20.4	917.5	1310.0
2-phenylmalonic acid	40.7	33.3	93.2	67.3	65.8	44.6	383.7	72.4	112.0	146.8
Unknown 033	144.3	90.0	0.0	0.0	102.7	23.2	304.3	126.9	112.6	51.3
p-cresol	82.0	46.6	43.5	60.2	97.8	30.7	96.6	43.8	163.5	431.9
2,6-dimethylpyrazine	0.0	267.8	0.0	0.0	86.3	22.3	188.8	181.8	0.0	0.0
Unknown 012	42.0	99.2	27.5	1.7	81.8	133.3	55.6	0.0	3029.1	2395.9
Unknown 030	68.1	82.5	50.2	36.8	135.2	37.7	151.0	263.0	91.6	139.6
butanoic acid	59.5	86.6	90.2	56.5	103.0	47.7	47.1	81.9	259.9	370.7
3-ethyl-2,5-dimethylpyrazine	111.0	111.6	2.5	120.2	82.6	20.0	238.8	122.8	1949.6	3010.6
Unknown 029	68.4	33.2	109.6	78.6	106.8	62.8	140.0	58.6	77.7	77.0
propanoic acid	57.9	81.8	139.0	86.9	80.2	38.2	116.4	52.6	186.6	171.7
Acetate	104.3	90.0	95.5	65.0	87.5	55.5	117.8	61.9	165.5	141.7
Indole	69.9	32.2	128.8	90.2	99.4	86.3	134.9	74.0	70.0	73.9
furaneol	97.1	85.1	143.4	53.8	78.6	54.9	262.2	149.2	181.4	119.5
3-methylbutanoic acid	58.2	39.5	173.8	121.3	69.2	51.3	84.3	34.4	228.3	270.3
tetradecanoic acid	101.7	104.4	156.7	38.1	116.0	2.5	168.2	60.1	140.5	141.0
isobutyric acid	55.3	48.1	183.2	117.9	73.3	48.9	48.8	23.0	212.0	228.4
4-methylpentanoic acid	33.3	63.8	88.9	71.8	256.7	61.4	139.0	15.2	181.3	124.7

**Table 4:** List of significant metabolites: comparison of baseline (B) and the 2nd week (W2) and their peak area values (Log_2_ transformed).

Metabolite	001_B	005_B	007_B	010_B	015_B	001_W2	005_W2	007_W2	010_W2	015_W2	*p*-Value	Pattern
**oleic acid**	26.24	26.56	27.35	26.88	27.19	27.38	27.99	27.65	28.50	28.13	0.0048	Up
**campesterol**	21.53	22.16	22.54	23.25	21.90	20.21	20.92	21.32	21.09	21.41	0.0076	Down
**stigmasterol**	21.48	20.63	20.75	21.86	21.06	20.17	N/A	20.10	19.81	20.56	0.0113	Down
lignoceric acid *	21.17	20.91	20.99	21.94	21.43	21.50	22.00	21.83	22.07	22.62	0.0248	Up
1-eicosanol *	23.06	20.75	20.28	23.03	22.01	19.97	19.97	20.49	19.16	20.81	0.0254	Down
**phosphate ion**	24.61	24.37	25.55	24.11	25.10	24.74	26.81	25.87	26.26	25.67	0.0312	Up
1-monolinolein *	21.56	22.50	22.64	21.70	22.05	22.41	22.93	22.32	22.88	23.18	0.0404	Up
**pimelic acid**	19.87	21.15	19.46	19.30	22.11	21.97	21.42	21.78	21.27	21.96	0.0490	Up
**maltose**	19.82	20.24	20.92	19.64	20.95	20.37	19.32	18.83	18.60	19.65	0.0494	Down
**L-cysteine**	20.97	21.22	20.77	21.31	22.76	21.75	24.69	23.15	22.23	22.19	0.0585	Up
**stearic acid**	22.72	23.86	23.65	24.28	23.32	22.32	22.96	23.31	22.85	23.01	0.0587	Down
**3-hydroxypyridine**	17.18	18.72	19.59	17.45	16.97	19.76	20.44	21.08	19.89	17.28	0.0706	Up
**methyl oleate**	21.96	22.40	23.10	22.88	21.08	20.87	24.64	24.61	25.62	25.39	0.0715	Up
**L-glutamic acid**	26.25	25.79	26.05	25.64	23.79	26.62	27.65	26.05	26.49	26.12	0.0734	Up
**N-acetyl-D-mannosamine**	22.94	24.14	24.32	25.04	23.36	22.55	20.95	23.72	23.19	23.34	0.0833	Down
**heptadecanoic acid**	23.77	24.25	23.90	26.11	24.01	23.27	23.42	24.06	22.90	23.75	0.0863	Down
**myristic acid**	24.76	24.77	24.39	25.79	24.69	24.16	23.84	25.08	23.59	24.32	0.0865	Down
2-oleoylglycerol *	20.83	20.88	21.44	20.83	21.34	20.90	21.78	21.16	22.25	21.85	0.0970	Up
**palmitic acid**	24.91	25.86	25.52	26.46	25.59	24.59	25.15	25.51	25.17	25.20	0.0998	Down

* confirmed only by matching with their mass fragmentation patterns to the databases.

**Table 5:** Proteomics results and their annotation.

	Proteins Identified	Human Proteins	% Non-Human Assigned EC	EC Families	EC Diversity (Inv Simpson)
**P001 B**	9768	550	51.00%	714	132
**P001 2**	10767	444	50.10%	707	130
**P001 12**	11545	371	50.62%	763	147
**P005 B**	5103	1353	38.53%	568	98
**P005 2**	6184	1749	37.44%	613	95
**P005 12**	10483	1441	43.91%	761	112
**P007 B**	11034	650	47.09%	740	103
**P007 2**	8289	559	44.46%	685	97
**P007 12**	9086	505	46.91%	679	107
**P010 B**	7975	585	48.54%	660	107
**P010 2**	11353	697	47.08%	774	120
**P010 12**	12109	718	46.73%	776	116
**P015 B**	3841	622	40.93%	551	89
**P015 2**	4797	672	40.84%	609	115
**P015 12**	6498	611	44.52%	655	110

## Supplementary Materials

The following are available online at https://www.mdpi.com/2072-6643/12/12/3749/s1, Supplementary Figure S1. PCA plots from volatile and global metabolomics data. All the peak area values obtained from metabolomics analysis were used in the principal component analysis and their clustering/distribution patterns were shown separately; volatile metabolites (left) and global metabolites (right). The severe scattering at the screening (baseline) time were reduced over the SCD treatment. Supplementary Data 1 Supplemental Metagenomic data.
